# Unraveling the Kinetics and Mechanism of Ethane Chlorination in the Gas Phase

**DOI:** 10.3390/molecules30081756

**Published:** 2025-04-14

**Authors:** Zihan Zhu, Yuting Li, Xia Wu, Jinming Xu, Xiaohui Sun, Qinggang Liu

**Affiliations:** 1 State Key Laboratory of Catalysis, Dalian Institute of Chemical Physics, Chinese Academy of Sciences, Dalian 116023, China; 2 University of Chinese Academy of Sciences, Beijing 100049, China; 3 Chemical Engineering and Resource Utilization, Northeast Forestry University, Harbin 150040, China; 4 State Key Laboratory of Heavy Oil Processing, China University of Petroleum (Beijing), Beijing 102249, China

**Keywords:** ethane chlorination, polyvinyl chloride, over-chlorination, chlorine radical

## Abstract

The selective chlorination of ethane to 1,2-dichloroethane offers a promising route for upgrading ethane, yet its efficiency remains constrained by limited mechanistic insights into gas-phase chlorine-radical-mediated pathways, which govern target product selectivity and competing dehydrochlorination side reactions. This work systematically decouples the kinetics of ethane chlorination and chloroethane functionalization under varying Cl_2_ concentrations, revealing that chlorine radicals govern product distribution through thermodynamically favored pathways. This results in an interesting phenomenon whereby the product ratio between 1,1-C_2_H_4_Cl_2_ and 1,2-C_2_H_4_Cl_2_ maintains a constant 2:1 stoichiometry regardless of Cl_2_ concentration variation. A critical observation is that the rate of all chlorination steps remains independent of alkane concentrations, highlighting the dominant role of chlorine radicals in rate-determining steps. Furthermore, ethylene byproducts are demonstrated to originate from the dechlorination of chlorine-radical-induced 2-chloroethyl radicals derived from chloroethane, rather than the direct dehydrochlorination of chloroethane itself. These insights into the dual role of chlorine radicals—mediating both the chlorination and dehydrochlorination pathways—establish a foundational framework for integrating gas-phase radical chemistry with catalytic engineering strategies to suppress undesired side reactions and enable scalable, selective ethane chlorination.

## 1. Introduction

As the second most abundant component of natural gas, ethane (C_2_H_6_) has great potential as a source for producing higher–value petrochemical feedstocks, such as light olefins, oxygenates, and aromatic hydrocarbons [1,2,3,4,5]. However, several challenges exist in C_2_H_6_ conversion. The C–H bonds in C_2_H_6_ are highly stable, with a dissociation energy of 423.2 kJ/mol [6,7,8]. Developing effective C–H activation methods is crucial for its utilization. Oxygen-assisted C–H bond dissociation has been studied to selectively synthesize ethylene (C_2_H_4_) from C_2_H_6_ at lower temperatures [9,10,11]. Yet, C_2_H_4_ is more reactive than C_2_H_6_, leading to inevitable over–oxidation [12,13,14]. Despite over 50 years of research on C_2_H_6_ oxidative dehydrogenation, selectivity limitations due to over–oxidation have made the industry rely on cracking methods. These endothermic methods, operating above 900 °C, are highly energy–intensive and produce large amounts of CO_2_ [15,16]. There is an urgent need to develop new low-temperature C_2_H_6_ conversion routes for producing chemical industry platform molecules, which is extremely important for the future of natural–gas-based chemistry [17,18].

Recently, Pérez-Ramírez and colleagues pioneered a catalytic process for the selective synthesis of 1,2-dichloroethane (1,2-C_2_H_4_Cl_2_) via C_2_H_6_ chlorination under mild conditions [19]. This innovative strategy holds transformative potential for large–scale C_2_H_6_ valorization, as 1,2-C_2_H_4_Cl_2_ serves as the primary precursor for commercial polyvinyl chloride (PVC)—a commodity chemical with an annual demand of 60 million tons, currently reliant on coal– and petroleum–derived feedstocks [20,21,22,23,24,25,26,27]. The downstream steps of this process leverage established industrial technologies, including cracking of 1,2-C_2_H_4_Cl_2_ to vinyl chloride (C_2_H_3_Cl) and HCl, Cl_2_ recovery from HCl via the Deacon process, and the polymerization of C_2_H_3_Cl [28,29]. Rare-earth oxyhalides exhibit exceptional structural stability in harsh (oxy)chlorination reactions, a property rooted in the thermodynamically unfavorable chlorination of rare-earth elements compared to transition metals under moderate-to-high temperatures [26,30]. Such robustness enables the retention of unsaturated metal cations with unpaired electrons on catalyst surfaces, which activate alkanes or alkyl halides while avoiding structural degradation into inactive chlorides [31,32,33]. These attributes have propelled rare-earth oxychlorides—particularly europium oxychloride—to the forefront of C_2_H_6_ chlorination, achieving 90% selectivity with stable performance over 40 h [19]. However, their industrial deployment remains limited by the necessity to operate at sub–20% C_2_H_6_ conversion and Cl_2_-lean concentrations to maintain high 1,2-C_2_H_4_Cl_2_ selectivity. Since its initial discovery by Pérez-Ramírez in 2021, this field has witnessed no substantive breakthroughs, highlighting the persistent challenges in efficiently converting C_2_H_6_ to target chlorocarbons.

**Figure 1:**
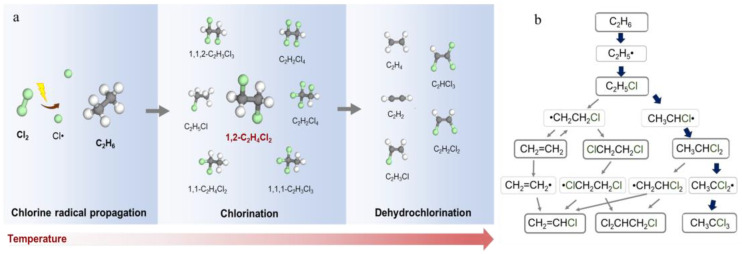
(**a**) Product distribution of the reaction between C_2_H_6_ and Cl_2_ in the gas−phase reaction pathway (Green spheres represent Cl atoms, gray spheres represent carbon atoms, and white spheres represent hydrogen atoms). (**b**) Potential reaction intermediates and pathways involved in ethane chlorination.

In the chlorination of C_2_H_6_, gas-phase free radicals pose a critical challenge to improving catalyst performance. Cl_2_, when thermally induced to dissociate, generates Cl radicals capable of abstracting hydrogen from C_2_H_6_ at low temperatures to form ethyl radicals [34]. These ethyl radicals can then react with Cl radicals to form chloroethane (C_2_H_5_Cl), offering a potential route for low-temperature C_2_H_6_ conversion [35,36]. However, hydrogen atoms in C_2_H_5_Cl can be further abstracted by Cl radicals, producing 1-chloroethyl and 2-chloroethyl radicals, with product distribution primarily governed by thermodynamic factors [37,38]. At high temperatures, these chlorinated alkyl radicals undergo dehydrogenation and further chlorination, forming complex reaction networks of competing chlorination and dehydrochlorination pathways (Figure 1a,b). Importantly, the dynamics of these elementary processes may vary under different temperatures and reactant atmospheres, but this knowledge remains incomplete. A thorough understanding of gas-phase chlorine radical behavior is critical for guiding subsequent catalyst design. Through the strategic coupling of gas-phase radicals with catalytic interfaces, we can achieve the selective synthesis of 1,2-dichloroethane (1,2-C_2_H_4_Cl_2_) while suppressing undesired side reactions—such as over-chlorination or dehydrochlorination—that arise from gas-phase chlorine radical pathways. In this study, we systematically examined the kinetics of key elementary steps—including C_2_H_6_ chlorination, C_2_H_5_Cl chlorination, and C_2_H_5_Cl dehydrochlorination-across varied temperatures and pressures. Our results demonstrate that all chlorination steps exhibit low dependence on alkane concentrations, confirming that chlorine radicals are the dominant species governing rate-determining hydrogen abstraction. Furthermore, mechanistic evidence reveals that ethylene byproducts predominantly form via the dechlorination of chlorine-radical-generated 2-chloroethyl radicals (derived from chloroethane intermediates) rather than through the direct dehydrochlorination of chloroethane. These findings elucidate the dual functionality of chlorine radicals in simultaneously mediating both the chlorination and dehydrochlorination pathways. These insights provide a foundational framework for strategically coupling gas-phase radical dynamics with catalytic engineering approaches, enabling the suppression of undesirable side reactions while achieving high 1,2-C_2_H_4_Cl_2_ selectivity at industrially relevant ethane conversion levels.

## 2. Results

### 2.1. Temperature-Dependent Ethane Chlorination

**Figure 2:**
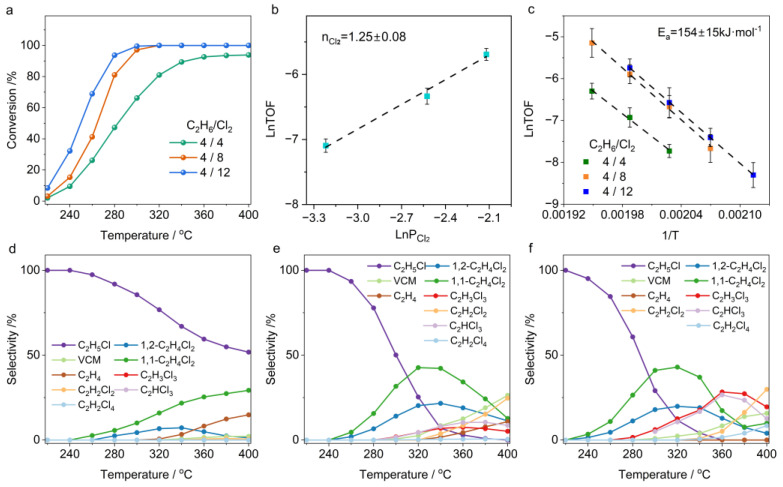
(**a**) Conversion as a function of temperature in C_2_H_6_ chlorination. (**b**) Reaction orders of Cl_2_. (**c**) Apparent activation energy of C_2_H_6_ chlorination. (**d**–**f**) Temperature-dependent product selectivity in C_2_H_6_ chlorination under different feed ratios: (**d**) C_2_H_6_:Cl_2_:N_2_ = 4:4:92 (vol.%); (**e**) C_2_H_6_:Cl_2_:N_2_ = 4:8:88 (vol.%); (**f**) C_2_H_6_:Cl_2_:N_2_ = 4:12:84 (vol.%).

The chlorination of C_2_H_6_ is a stepwise process, and the ratio of C_2_H_6_ to chlorine (Cl_2_) has a critical impact on the product distribution. In our experimental design, we maintained a fixed ethane concentration of 4 vol.% in the feed gas while systematically investigating conversion efficiency and product distribution patterns across varying Cl_2_ concentration gradients (4–12 vol.%). Figure 2a shows a clear linear correlation between C_2_H_6_ conversion rates and Cl_2_ concentrations. Increasing the Cl_2_ partial pressure significantly enhances C_2_H_6_ conversion, particularly under low-temperature conditions. For example, at 240 °C, when the Cl_2_ concentration increases from 4 vol.% to 12 vol.%, the C_2_H_6_ conversion rate sharply rises from 10% to 33%. This critical role of Cl_2_ in the reaction is further supported by its reaction order, which can reach as high as 1.25 according to kinetic studies (Figure 2b). Interestingly, our experiments revealed that altering the Cl_2_ concentration does not significantly affect the activation energy for C_2_H_6_ conversion, indicating that the reaction mechanism remains unchanged despite variations in Cl_2_ concentration (Figure 2c). These results suggest that Cl_2_ concentration plays a vital role in promoting chlorine radical proliferation in the gas phase, making it a key and effective method for achieving high-efficiency C_2_H_6_ conversion under low-temperature conditions.

At temperatures below 240 °C, the products of C_2_H_6_ chlorination are primarily C_2_H_5_Cl (with selectivity exceeding 90%) and are minimally influenced by Cl_2_ partial pressure. This indicates that the further chlorination of C_2_H_5_Cl requires a higher activation energy barrier, consistent with chlorinated alkane reactivity decreases with increasing chlorine substitution [35]. Increasing the reaction temperature significantly promotes the further chlorination of C_2_H_5_Cl, but the product distribution is determined by the Cl_2_ concentration. Under the reaction condition of C_2_H_6_/Cl_2_ at a 4:4 ratio, as the temperature rises, the further chlorination of C_2_H_5_Cl is gradually initiated (Figure 2d). In this process, 1,1-C_2_H_4_Cl_2_ and 1,2-C_2_H_4_Cl_2_ are almost simultaneously generated, but the formation rate of 1,1-C_2_H_4_Cl_2_ is approximately twice that of 1,2-C_2_H_4_Cl_2_. Increasing the Cl_2_ partial pressure significantly enhances the chlorination of C_2_H_5_Cl, shifting the generation curve of dichloroethane to lower temperatures. Notably, the product ratio between 1,1-C_2_H_4_Cl_2_ and 1,2-C_2_H_4_Cl_2_ maintains a constant 2:1 stoichiometry regardless of Cl_2_ concentration variation. This phenomenon originates from the regioselective nature of chlorine radical (Cl·)–mediated hydrogen abstraction in gas-phase chlorination. During C_2_H_5_Cl chlorination, Cl· preferentially abstracts α–H from the chlorine-substituted carbon atom (C–Cl site), generating the thermodynamically favored 1-chloroethyl radical intermediate, which is approximately 15 kJ/mol more stable than the 2-chloroethyl isomer [37,39].

It is noteworthy that under Cl_2_-lean conditions (C_2_H_6_/Cl_2_ = 4/4), elevated temperatures enhance C_2_H_6_ conversion but deplete Cl_2_ in the feedstock, thereby suppressing further chlorination of C_2_H_5_Cl and favoring its decomposition to C_2_H_4_ (Figure 2d). Our analysis of product distribution reveals that during C_2_H_5_Cl chlorination, both 1-chloroethyl and 2-chloroethyl radicals are involved. The latter undergoes preferential dechlorination, serving as the primary precursor for ethylene formation. This mechanistic insight explains the experimental observation where C_2_H_4_ generation coincides with the disappearance of 1,2-C_2_H_4_Cl_2_ (Figure 2d). Under stoichiometric conditions (C_2_H_6_/Cl_2_ = 4/8), C_2_H_5_Cl readily undergoes chlorination to form dichloroethane. At temperatures exceeding 300 °C, the simultaneous decomposition of 1,1-C_2_H_4_Cl_2_ and 1,2-C_2_H_4_Cl_2_ is activated, accompanied by C_2_H_3_Cl production. Further increasing the temperature to 320 °C triggers the decomposition of trichloroethane (C_2_H_3_Cl_3_) and tetrachloroethane (C_2_H_2_Cl_4_), leading to the significant formation of dichloroethylene (C_2_H_2_Cl_2_) and trichloroethylene (C_2_HCl_3_) as byproducts. Conversely, under Cl_2_-rich conditions (C_2_H_6_/Cl_2_ = 4/12), temperature elevation predominantly induces the over-chlorination of dichloroethane, while its decomposition is markedly suppressed. Notably, tetrachloroethane (C_2_H_2_Cl_4_) exhibits extreme thermal instability above 300 °C, decomposing to generate C_2_HCl_3_. Consequently, C_2_H_3_Cl_3_ and C_2_HCl_3_ dominate the byproduct profile under Cl_2_-rich environments.

**Figure 3:**
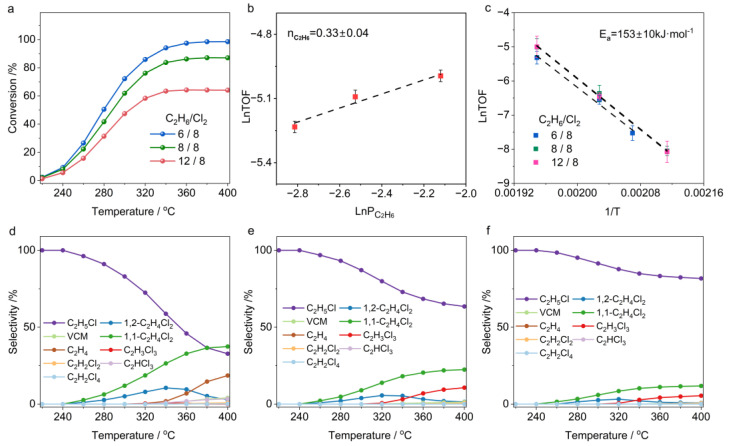
(**a**) Conversion as function of temperature in C_2_H_6_ chlorination. (**b**) Reaction orders of C_2_H_6_. (**c**) Apparent activation energy of C_2_H_6_ chlorination. (**d**–**f**) Temperature-dependent product selectivity in C_2_H_6_ chlorination under different feed ratios: (**d**) C_2_H_6_:Cl_2_:N_2_ = 6:8:86; (**e**) C_2_H_6_:Cl_2_:N_2_ = 8:8:84; (**f**) C_2_H_6_:Cl_2_:N_2_ = 12:8:80.

These experimental results demonstrate that under high-temperature conditions, chlorinated products undergo further chlorination and dehydrochlorination is governed by the Cl_2_ concentration. These findings prompted us to further investigate the impact of C_2_H_6_ concentration on product distribution by maintaining a fixed Cl_2_ partial pressure of 8 vol.%. The results are shown in Figure 3. As indicated in Figure 3a, unlike Cl_2_, increasing C_2_H_6_ partial pressure significantly reduces the equilibrium conversion of C_2_H_6_. The kinetic analysis reveals that changes in C_2_H_6_ concentration have little effect on the intrinsic conversion frequency (Figure 3b). This suggests that the chlorination of C_2_H_6_ is primarily driven by chlorine radicals. This conclusion is further supported by the results in Figure 3c, which show that altering C_2_H_6_ concentration has minimal impact on the activation energy for C_2_H_6_ chlorination.

Across all experimental conditions examined, the 1,1-C_2_H_4_Cl_2_ isomer exhibited a consistent twofold formation rate advantage over the 1,2-C_2_H_4_Cl_2_ isomer. This observation provides additional evidence that the chlorine-radical-mediated substitution of α–H in C_2_H_5_Cl proceeds exclusively through a thermodynamically controlled reaction pathway. When the ratio of C_2_H_6_ to Cl_2_ was 6:8, we again observed, at temperatures above 320 °C, that C_2_H_4_ production was negatively correlated with the formation of 1,2-C_2_H_4_Cl_2_ (Figure 3d). This suggests that 2-chloroethyl radicals are unstable and tend to decompose rather than further chlorinate to form 1,2-C_2_H_4_Cl_2_ at high temperatures. The comparative analysis of the 8:8 versus 4:4 feed ratios reveals an interesting trend: despite maintaining stoichiometric equivalence (C_2_H_6_:Cl_2_ = 1:1), elevated Cl_2_ partial pressure promotes progressive chlorination of C_2_H_5_Cl, thereby inducing competitive Cl_2_ consumption (Figure 2d and Figure 3e). This cascade effect ultimately suppresses ethane conversion under the 8:8 condition. Unexpectedly, under Cl_2_-lean conditions where the C_2_H_6_-to-Cl_2_ ratio is less than 1, the decomposition of C_2_H_5_Cl is well-suppressed even when the temperature is increased to 400 °C (Figure 3f). In contrast, under Cl_2_-rich conditions (C_2_H_6_-to-Cl_2_ ratio of approximately 1:2), the decomposition of C_2_H_5_Cl begins at 320 °C and significantly intensifies with increasing temperature. This indicates that C_2_H_4_ byproducts are not directly generated through the thermal decomposition of C_2_H_5_Cl, but rather, originate from 2-chloroethyl radicals, which will be discussed in detail in Section 2.2.

### 2.2. Temperature-Dependent Chloroethane Chlorination

**Figure 4:**
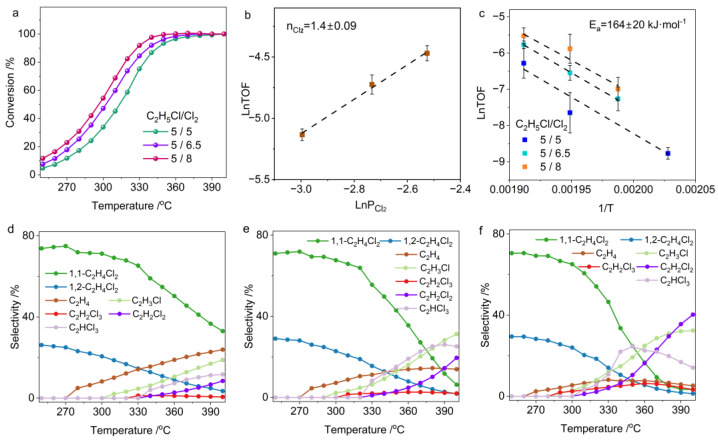
(**a**) Conversion as function of temperature in C_2_H_5_Cl chlorination. (**b**) Reaction orders of Cl_2_. (**c**) Apparent activation energy of C_2_H_5_Cl chlorination. (**d**–**f**) Temperature-dependent product selectivity in C_2_H_5_Cl chlorination under different feed ratios: (**d**) C_2_H_5_Cl:Cl_2_:N_2_ = 5:5:90; (**e**) C_2_H_5_Cl:Cl_2_:N_2_ = 5:6.5:88.5; (**f**) C_2_H_5_Cl:Cl_2_:N_2_ = 5:8:87.

As demonstrated by the experiments, the chlorination of C_2_H_6_ proceeds stepwise, initially forming C_2_H_5_Cl, followed by subsequent chlorination or dehydrochlorination side reactions. To further elucidate the factors governing the reaction kinetics of C_2_H_5_Cl, we systematically investigated its reactivity under varying Cl_2_ concentrations (5–8 vol.%) while maintaining a fixed C_2_H_5_Cl concentration of 5 vol.%. As illustrated in Figure 4a, the light–off curves demonstrate that the chlorination of C_2_H_5_Cl is highly dependent on Cl_2_ concentration, with the onset temperature for chlorination shifting significantly toward lower temperatures as the Cl_2_ concentration increases. This observation aligns with the kinetic analysis results, which revealed a reaction order of 1.4 for Cl_2_, highlighting its pivotal role in promoting further chlorination of C_2_H_5_Cl (Figure 4b). Notably, the higher reaction temperature required for chloroethane chlorination compared to ethane chlorination to chloroethane could also be attributed to its smaller kinetic constant, as increasing the chlorine concentration has minimal impact on the activation energy of chloroethane chlorination (164 ± 20 vs. 154 ± 15 kJ/mol) but significantly enhances the low-temperature conversion rate.

In all ranges of Cl_2_ concentration, the initial chlorination products of C_2_H_5_Cl are primarily 1,1-C_2_H_4_Cl_2_, which is twice the amount of 1,2-C_2_H_4_Cl_2_. Interestingly, the product distribution at high temperatures is significantly influenced by Cl_2_ concentration. Under Cl_2_-lean conditions (C_2_H_5_Cl/Cl_2_ = 5/5 *vol.*/*vol.*), C_2_H_4_ and C_2_H_3_Cl as byproducts are observed at 300 °C, and their formation is significantly enhanced with increasing temperature, accompanied by the substantial consumption of dechlorinated products (Figure 4d). In contrast, under Cl_2_-rich conditions (C_2_H_5_Cl/Cl_2_ = 5/8), high temperatures induced the overchlorination of dichloroethane, resulting in products primarily consisting of trichloro and tetrachloroethanes (Figure 4f). These compounds further decomposed to form byproducts such as dichloroethylene and trichloroethylene. These phenomena demonstrate that dehydrohalogenation side reactions of chlorinated alkanes in the presence of Cl_2_ are unavoidable. The resulting unsaturated chlorinated hydrocarbons can remain stable under a Cl_2_ atmosphere. This poses a significant challenge for designing efficient catalysts targeting selective C_2_H_6_ chlorination to 1,2-C_2_H_4_Cl_2_, as such unsaturated compounds readily induce catalyst poisoning and deactivation.

**Figure 5:**
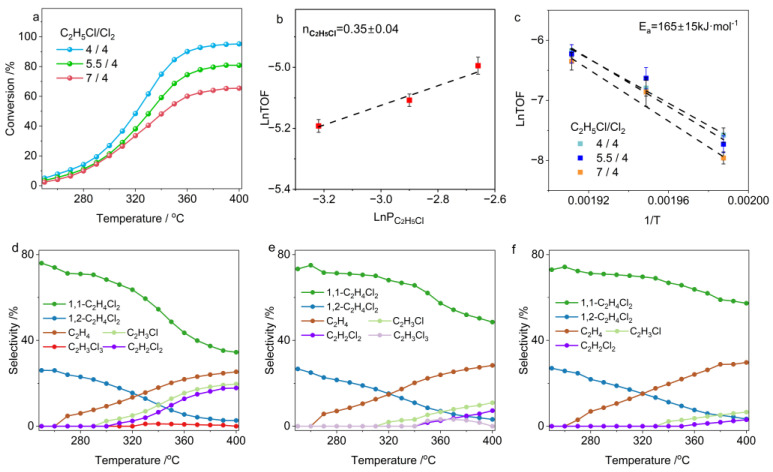
(**a**) Conversion as function of temperature in C_2_H_5_Cl chlorination. (**b**) Reaction orders of C_2_H_5_Cl. (**c**) Apparent activation energy of C_2_H_5_Cl chlorination. (**d**–**f**) Temperature-dependent product selectivity in C_2_H_5_Cl chlorination under different feed ratios: (**d**) C_2_H_5_Cl:Cl_2_:N_2_ = 4:4:92; (**e**) C_2_H_5_Cl:Cl_2_:N_2_ = 5.5:4:90.5; (**f**) C_2_H_5_Cl:Cl_2_:N_2_ = 7:4:89.

With the Cl_2_ concentration fixed at 4 vol.%, we further investigated the reaction kinetics of C_2_H_5_Cl chlorination under varying C_2_H_5_Cl concentrations. Figure 5a shows that higher C_2_H_5_Cl concentrations significantly reduce the equilibrium conversion, yet have a limited effect on the low-temperature conversion. This behavior stems fundamentally from the stoichiometric effect—specifically, excess reactant ratios (e.g., higher Cl_2_/C_2_H_5_Cl) will thermodynamically favor higher conversion of the C_2_H_5_Cl reactant. Kinetic studies indicate a reaction order of 0.35 for C_2_H_5_Cl (Figure 5b), further confirming that Cl_2_ concentration is the main governing factor in the chlorination process. Combined with kinetic data from C_2_H_6_ chlorination to C_2_H_5_Cl, these results conclusively demonstrate that the overall rate of C_2_H_6_ chlorination to dichloroethane is primarily dictated by Cl_2_ concentration. An Arrhenius analysis further confirmed that variations in C_2_H_5_Cl concentration exerted negligible effects on the activation energy of C_2_H_5_Cl chlorination (Figure 5c).

The chlorination product distribution of C_2_H_5_Cl exhibits pronounced concentration dependence and temperature-responsive behavior. At low temperatures, the primary products of C_2_H_5_Cl chlorination are 1,1-C_2_H_4_Cl_2_ and 1,2-C_2_H_4_Cl_2_. However, at high temperatures, the product distribution is governed by the C_2_H_5_Cl-to-Cl_2_ feed ratio. At a C_2_H_5_Cl/Cl_2_ ratio of 4/4 (Figure 5d), as the temperature rises to 280 °C, the dehydrochlorination of C_2_H_5_Cl is significantly activated. Meanwhile, dichloroethane tends to convert into trichloroethane, accompanied by dehydrochlorination, and by-products like dichloroethylene can be detected in the exhaust gas. Interestingly, when the Cl_2_ concentration is reduced (Figure 5e,f), the decomposition of C_2_H_5_Cl to ethylene is almost unaffected, but the over-chlorination and decomposition of dichloroethane are markedly suppressed, with this inhibition intensifying as Cl_2_ concentration further decreases. This indicates that hydrogen on C_2_H_5_Cl is more easily abstracted by chlorine radicals than that on polychlorinated alkanes. This higher reactivity of C_2_H_5_Cl allows it to act as a competitive molecule, inhibiting the further conversion of dichloroethane (including over-chlorination or dehydrochlorination). Maintaining a certain amount of C_2_H_5_Cl in the atmosphere can enhance dichloroethane selectivity. However, the decomposition of C_2_H_5_Cl under a Cl_2_ atmosphere presents new challenges for designing C_2_H_6_ chlorination catalysts. Specifically, reducing the Cl_2_ concentration may suppress the over-chlorination of dichloroethane, but the Cl_2_ concentration must be carefully optimized. This is because dehydrochlorination side reactions of C_2_H_5_Cl will significantly increase, producing unsaturated hydrocarbons, which have a critical impact on the lifespan of the catalyst.

**Figure 6:**
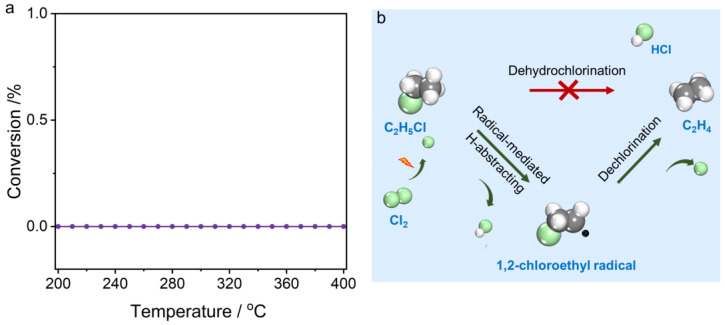
(**a**) Thermal decomposition profile of C_2_H_5_Cl under C_2_H_5_Cl/N_2_ feed ratio of 9/91. (**b**) Proposed mechanism of chlorine−radical−mediated decomposition of C_2_H_5_Cl to ethylene.

Notably, the thermal decomposition of C_2_H_5_Cl is markedly inhibited in chlorine-free environments, even at elevated temperatures (400 °C, Figure 6a). This phenomenon clearly demonstrates that ethyl chloride itself exhibits excellent stability under the tested conditions. This behavior originates from the intrinsic bond strength of its carbon–chlorine (C–Cl) and carbon–hydrogen (C–H) bonds. In the absence of an external reactive species, the energy provided by the temperature of 400 °C is not sufficient to break these bonds. Conversely, when Cl_2_ is introduced, Cl_2_ molecules absorb energy and dissociate into highly reactive chlorine radicals. These radicals exhibit strong electrophilicity due to their unpaired electrons. Upon encountering C_2_H_5_Cl molecules, they abstract a hydrogen atom from C_2_H_5_Cl, mediating the formation of 2-chloroethyl radicals.

Under elevated temperatures in chlorine-lean environments, these intermediates predominantly undergo dechlorination to yield ethylene rather than participating in further chlorination pathways to produce 1,2-C_2_H_4_Cl_2_ (Figure 6b). In Cl_2_-lean conditions, insufficient Cl_2_ molecules are available for the 2-chloroethyl radicals to engage in sequential chlorination. Instead, the 2-chloroethyl radicals undergo intramolecular electronic rearrangement, which weakens the α–C–Cl bond. This facilitates the loss of a chlorine atom, yielding an ethylene molecule and regenerating a chlorine radical. Thermodynamically, this pathway is favored under these conditions due to the significant energy release associated with ethylene formation. This understanding of chlorine-radical-induced dechlorination reactions is crucial for optimizing reaction conditions and designing superior C_2_H_6_ chlorination catalysts.

## 3. Materials and Methods

**Figure 7:**
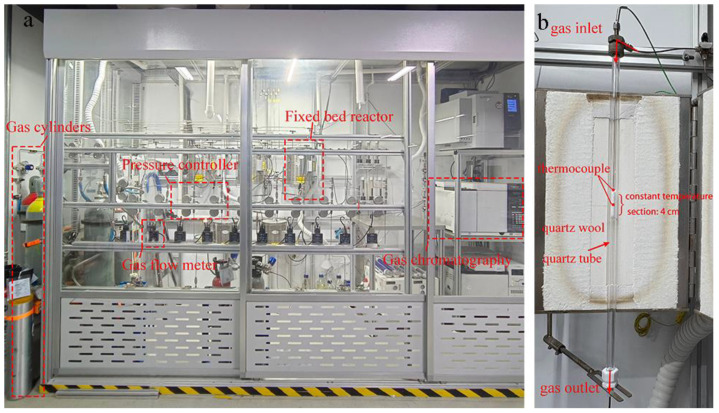
(**a**) A photograph of the ethane chlorination set−up. (**b**) A detailed view of the reactor.

The catalytic tests were performed at ambient pressure in a continuous-flow fixed-bed reactor set-up (Figure 7). The quartz tubular reactor (internal diameter d_i_ = 8 mm, L = 450 mm) was loaded and placed in an electrical oven. The furnace maintained an isothermal zone (±1 °C) over a 40 mm length. To ensure efficient heating of the feed gas, the quartz tube was packed with a 40 mm layer of quartz wool, as illustrated in Figure 7. A K-type thermocouple fixed in a coaxial quartz thermowell with the tip positioned in the center of the bed was used to monitor the temperature during the reaction. The bed was then heated in the reaction gas (15 mL/min) to the desired temperature (200–400 °C) and stabilized for at least 30 min, and the reaction tail gas was subsequently analyzed. Unless otherwise specified, all thermal treatment processes were carried out with a heating rate of 5 K·min^−1^.

The gases C_2_H_6_, C_2_H_5_Cl, Cl_2_, and N_2_ (carrier gas) were fed by digital mass flow controllers (Bronkhorst^®^, Veenendaa, The Netherlands) to a mixing unit. The effluent gas stream was neutralized by passing it through an impinging bottle containing a saturated NaOH solution. The content of the carbon–containing compounds (C_2_H_6_, C_2_H_4_, and their chlorinated derivatives) in the reactor-outlet gas stream were analyzed online by an Agilent 7890B gas chromatograph (GC, manufactured in Santa Clara, CA, USA) equipped with an HP-PLOT Q capillary column and an FID detector. For highly chlorinated products (e.g., C_2_H_2_Cl_2_, C_2_HCl_3_), additional structural identification was performed by gas chromatography coupled with quadrupole time-of-flight high-resolution mass spectrometry (GC-QTOF HRMS, Agilent7250GC/Q-TOF, manufactured in Santa Clara, CA, USA). All kinetic parameters (e.g., activation energy and reaction orders) were derived from reaction rates measured at low conversion levels (<10%) to ensure a nearly constant reaction rate in the entire reactor volume.

The conversion of reactant i, X(i) (i: C_2_H_6_, C_2_H_5_Cl), was calculated from the following equation:

$$X\left(i\right)=\frac{n{\left(i\right)}^{\mathrm{inlet}}-n{\left(i\right)}^{\mathrm{outlet}}}{n{\left(i\right)}^{\mathrm{inlet}}}\cdot 100\%$$

where n(i)^inlet^ and n(i)^outlet^ are the molar flows of the reactant i at the inlet and outlet of the reactor, respectively. The selectivity (S(j)) of product j (j: C_2_H_4_, C_2_H_5_Cl, C_2_H_3_Cl, 1,1-C_2_H_4_Cl_2_, 1,2-C_2_H_4_Cl_2_, and 1,1,2-C_2_H_3_Cl_3_, etc.) was determined from the following equation:

$$S\left(j\right)=\frac{n{\left(j\right)}^{\mathrm{outlet}}\cdot N_{C}\left(j\right)}{\sum n{\left(j\right)}^{\mathrm{outlet}}\cdot N_{C}\left(j\right)}\cdot 100\%$$

where n(j)^outlet^ is the molar flow of the product j at the reactor outlet, and N_C_(j) is the number of carbon atoms in the compound j.

The reaction order is typically determined experimentally using the rate expression:

$$r=k\cdot {\left[A\right]}^{m}{\left[B\right]}^{n}$$

where r is the reaction rate, k is the rate constant, and m and n represent the partial reaction orders with respect to reactants A and B, respectively. The initial reaction rate is measured by varying the initial concentration of one reactant while keeping the concentrations of the other reactants constant. The reaction order for a specific reactant is the slope derived from the logarithmic relationship:

$$\ln r=\ln k+m\cdot \ln\left[A\right]+n\cdot \ln\left[B\right]$$

The activation energy (Ea) is calculated using the Arrhenius equation:

$$k=A\cdot e^{-E_{a}/\left(RT\right)}$$

where k is the rate constant, A is the pre-exponential factor, R is the gas constant (8.314 J·mol^−1^·K^−1^), and T is the temperature in Kelvin. By measuring rate constants (k) at multiple temperatures, Ea can be determined from the linearized form:

$$\ln k=\ln A-\frac{E_{a}}{R}\times \frac{1}{T}$$

A plot of lnk versus 1/T (Arrhenius plot) yields a straight line with slope (−Ea/R), allowing Ea to be derived.

In a real experiment, both reaction orders and activation energies were calculated based on the average turnover frequencies (TOF) obtained at low conversion levels. The turnover frequency (TOF) can be calculated using the following formula:

$$TOF=\frac{C_{feed}\cdot F_{gas}\cdot X\cdot t}{n_{active\;sites}\cdot \;t}=\frac{C_{feed}\cdot F_{gas}}{n_{active\;sites}}\;\cdot X$$

where C_feed_ is the feed gas concentration, F_gas_ is the volumetric gas flow rate, X is the conversion of the reactant, t is the reaction time, n_active sites_ is the total moles of catalytic active sites.

The active sites on the catalyst surface (here, quartz wool) remain consistent across all experiments, since the same reactor configuration was employed. Additionally, the gas flow rate was intentionally increased when necessary to ensure conversions remained below 10%.

If the gas flow rate (F_gas_) remains constant, the terms C_feed_, F_gas_, and n_active sites_ remain identical across all experimental conditions. Consequently, whether these parameters are explicitly included in the TOF calculation does not affect the slopes of lnTOF ~ lnP (for reaction order) or lnTOF ~ 1/T (for Ea), as they cancel out mathematically.

When F_gas_ varies between experiments, the TOF formula must explicitly account for flow rate:

$$TOF=\frac{C_{feed}}{n_{active\;sites}}\cdot F_{gas}\cdot \;X$$

## 4. Conclusions

In conclusion, this study systematically investigates the chlorination and dehydrochlorination of C_2_H_6_ under varying C_2_H_6_/Cl_2_ molar ratios and reaction conditions. The results demonstrate that the C_2_H_6_/Cl_2_ molar ratio significantly influences the reaction pathways, with lower Cl_2_ concentrations favoring the formation of C_2_H_5_Cl and higher concentrations promoting further chlorination to dichloroethane. Additionally, the study reveals that the dehydrochlorination of C_2_H_5_Cl is fundamentally driven by the excitation of chlorine radicals, with the 2-chloroethyl radical serving as a critical precursor for ethylene formation. These findings underscore the importance of optimizing the C_2_H_6_/Cl_2_ molar ratio to suppress undesired side reactions and enhance the selectivity of target chlorocarbons. These insights provide a theoretical foundation for designing efficient catalysts and reaction conditions to achieve selective C_2_H_6_ chlorination to 1,2-C_2_H_4_Cl_2_, a key precursor for polyvinyl chloride production.

## Data Availability

The original contributions presented in this study are included in the article. Further inquiries can be directed to the corresponding authors.
